# Le sujet âgé en hémodialyse chronique: expérience d'un centre hospitalier marocain

**DOI:** 10.11604/pamj.2013.15.25.2278

**Published:** 2013-05-15

**Authors:** Ilham Karimi, Nawal Benabdellah, Yassamine Bentata, Intissar Haddiya

**Affiliations:** 1 Service de Néphrologie-Dialyse, Hôpital Al farabi, Université Mohamed Premier, Faculté de médecine et de pharmacie, Oujda, Maroc

**Keywords:** Sujet âgé, hémodialyse, insuffisance rénale chronique, comorbidité cardiovasculaire, troubles cognitifs, elderly, hemodialysis, chronic renal failure, cardiovascular comorbidity, cognitive impairment

## Abstract

De plus en plus de patients âgés sont pris en charge en hémodialyse chronique. L'augmentation de l'incidence de la maladie rénale chronique, du diabète et l'amélioration de la survie cardio-vasculaire expliquent en grande partie ce constat. Les conséquences de la prise en charge de ces patients âgés en en hémodialyse chronique sont multiples. Elles sont dominées par les comorbidités et les troubles d'autonomie. Le but de notre étude est de décrire le profil épidémiologique et clinique des patients âgés traités par HDC dans notre centre. Nous avons réalisé une étude transversale, en Novembre 2011, incluant tous les patients hémodialysés chroniques de l'Hôpital Al Farabi d'Oujda, dans la région de l'Oriental Marocain. Nous avons analysé chez les patients âgés de 65 ans et plus les paramètres démographiques, clinico-biologiques et dialytiques. L'analyse statistique est réalisée par le logiciel SPSS 17,0. Parmi 94 patients hémodialysés chroniques actuellement pris en charge dans notre centre, 31patients sont âgés de 65 ans et plus (32,9%). La néphropathie causale était diabétique dans 6,4% des cas, vasculaire dans 19,35% des cas, et glomérulaire dans 20% des cas. Les comorbidités sont observées chez 65% de ces patients. 29% de patients avaient une comorbidité cardiovasculaire, 55% étaient hypertendus, alors que 6,4% avaient des troubles cognitifs. Les sujets âgés en hémodialyse chronique représentent une population de patients fragiles. Ils nécessitent une surveillance particulière et régulière afin de prévenir certaines complications propres au sujet âgé et améliorer ainsi leur qualité de vie.

## Introduction

Au Maroc, l'effectif des personnes âgées de 60 ans et plus s'est élevée de 833.000 à 2,4 millions entre 1960 et 2010, soit une augmentation annuelle de 2,3% [1]. Le vieillissement de la population et l'augmentation de l'espérance de vie nous ont conduit à observer un plus grand nombre de sujets âgés dans toutes les disciplines médicales et en particulier en néphrologie où l'incidence des atteintes rénales a tendance à s'accroître d'année en année et constitue par conséquent un problème de santé publique.

Ainsi, de plus en plus de patients âgés sont pris en charge en hémodialyse chronique (HDC). L'augmentation de l'incidence de la maladie rénale chronique, du diabète et l'amélioration de la survie cardio-vasculaire expliquent en grande partie ce constat [2, 3]. Les conséquences de la prise en charge de ces patients âgés en HDC sont multiples. Elles sont dominées par les comorbidités et les troubles d'autonomie [2, 3].

Par ailleurs, les problèmes soulevés par la dialyse chez le sujet âgé sont multiples et intriqués, à la fois matériels (techniques, pratiques, logistiques), éthiques et économiques [4]. L'objectif de notre étude était de décrire le profil épidémiologique et clinique des patients âgés traités par HDC dans notre centre.

## Méthodes

Nous avons réalisé une étude transversale, en Novembre 2011, incluant tous les patients hémodialysés chroniques de l'Hôpital Al Farabi d'Oujda, dans la région de l'Oriental Marocain. Pour l'organisation mondiale de la santé (OMS), une personne est définie comme âgée si elle est âgée de 65 ans ou plus. Nous avons analysé chez nos patients les paramètres suivants:

**Démographiques** à savoir l'âge, le sexe, le niveau socio-économique; **cliniques:** Antécédents médico-chirurgicaux, la néphropathie initiale, les comorbidités (Hypertension artérielle (HTA) définie par une pression artérielle systolique> 140 mmhg et/ou une pression artérielle diastolique > 90mmhg; Diabète; Maladie cardio-vasculaire; néoplasie; Maladie de système évolutive); **Biologiques:** Hémoglobine (L'anémie définie par une baisse de l'hémoglobine (Hb) en dessous de 11 g/dl). Bilan phospho-calcique (calcémie, phosphorémie, PTH intacte, 25 OHvitamine D), bilan lipidique (cholestérol total, triglycérides); **Dialytiques:** ancienneté en HDC, nombre de séance / semaine. Nos patients sont tous dialysés par des générateurs 4008 (Fresenius). Le Bain de dialyse utilisé est un bain standard, le tampon utilisé est le bicarbonate, avec une teneur en calcium à 1,75mmol/l, potassium à 2meq/l, et Glucose à 0, 3g/l. **Thérapeutique:** les différents traitements en cours (traitement antihypertenseur, agents stimulants de l'érythropoïèse, supplémentation par le fer, calcium, chélateur de phosphore, analogues de la vitamine D).

L'analyse statistique a été réalisée par le logiciel SPSS 17.0. Les variables quantitatives sont exprimées en moyenne ± écart-type et les variables qualitatives en pourcentages. Une valeur p < 0,05 est considérée significative.

## Résultats

### Données démographiques, clinico-biologiques et dialytiques de nos patients HDC

Parmi 94 patients hémodialysés chroniques actuellement pris en charge dans notre centre. 31 patients sont âgés de 65 ans et plus (32,9%). avec une prédominance féminine (10H,21F). L'âge moyen de ces patients était de 69,2±4,15 ans [65-85 ans]. 56,25% de nos patients appartiennent à un faible niveau socio-économique.

**Figure 1:**
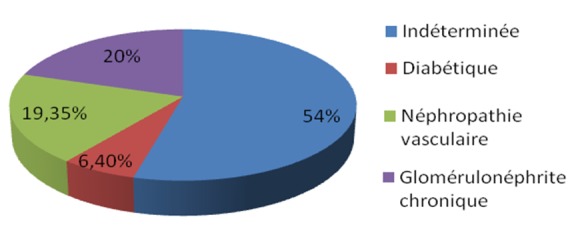
Néphropathies causales chez nos patients hémodialysé chronique

La néphropathie causale était diabétique dans 6,4% des cas, vasculaire dans 19,35% des cas, et glomérulaire dans 20% des cas. Elle était indéterminée (NI) dans 54% (Figure 1). 29% de patients avaient une comorbidité cardiovasculaire, 55% étaient hypertendus, alors que 6,4% avaient des troubles cognitifs.

Sur l'ensemble de nos patients âgés, 65% déclarent n'avoir aucune activité physique. Il s'agit essentiellement des patients assistés, dans leurs activités quotidiennes, par une tierce personne.

Sur le plan biologique 80% des patients étaient anémiques, avec une hémoglobine moyenne à 8,8 + /1,8. L'hypercholestérolémie était présente chez 22% d'entre eux. Les troubles du métabolisme minéral et osseux étaient observés dans 71,8% des cas. Il s'agissait d'hyperparathyroidie secondaire dans 62,5% des cas, hypoparathyroidie dans 6,2% des cas et ostéomalacie dans 3,1% des cas. Le traitement de nos patients était limité à la supplémentation calcique ainsi que la chélation du phosphore basé sur les chélateurs calciques, qui étaient utilisés dans 56,2% des cas. Le Tableau 1 résume les données biologiques.

**Tableau 1:** Paramètres biologiques sanguins

Paramètres biologiques sanguins	Moyenne ± Ecart-type (ET)
glycémie (g/l)	1,43±1,3
Hémoglobine (g/dl)	9,8± 1,8
PTH1-84 (pg/l)	508± 380
Calcémie (mg/l)	85,06±12,02
Phosphorémie (mgl)	61,16 ±10,44
25 OH Vitamine D	23,9 ± 6,2
Phosphatases alcalines (UI/l)	87,8 ±18
Cholestérol total	2,01 ± 0,9
Triglycérides	1,90 ± 0,74

Sur le plan thérapeutique, 55% des patients recevait un traitement antihypertenseur, 33% des patients étaient sous ASE. L'alfacalcidiol était prescrite chez 40,9% des patients, la vitamine D native était prescrite chez 3% des patients.

Concernant les données dialytiques de nos patients, la durée moyenne de dialyse était de 10,48±6,23 ans. 35% de nos patients avaient un rythme de 2 séances par semaine, tandis que les 65% restants étaient dialysés à raison de 3 fois par semaine.

## Discussion

La prévalence de l'IRT en suppléance progresse de façon linéaire dans tous les pays développés et cette progression concerne principalement les sujets âgés, aussi bien aux Etats-Unis et au Canada qu'en Europe [5]. Dans notre service, les Sujet âgés en hémodialyse représentent 32% de nos malades.

Les néphropathies initiales chez les hémodialysés âgés sont nettement dominées par les néphropathies vasculaires. Il est également noté un taux considérable de diabète non insulinodépendant, souvent compliqué de lésions vasculaires. Enfin, les néphropathies interstitielles, le plus souvent de cause obstructive, occupent une place variable selon les publications [6]. Dans notre série la néphropathie initiale était vasculaire dans 19.38%, alors qu'elle était indéterminée chez 54% des patients.

La population âgée en hémodialyse se caractérise par une fréquence élevée de comorbidités, tout particulièrement les coronaropathies, les artériopathies périphériques, l'insuffisance cardiaque congestive, le diabète et l'hypertension artérielle [4].

Ces comorbidités viennent largement en tête des causes de morbi-mortalité et d'hospitalisations, leur incidence augmente aussi avec l'âge et l'ancienneté en hémodialyse [7, 8]. Leur prise en charge précoce s'impose en même temps que la néphroprotection.

Dans notre étude 29% de patients avaient une comorbidité cardiovasculaire et 55% étaient hypertendus sous traitement antihypertenseur.

La dégradation des fonctions cognitives est l'apanage des patients âgés, les désordres allant de simples troubles de la mémoire à une démence profonde. La prévalence semble augmentée chez les hémodialysés, Quatre études en centre de dialyse montraient que 24 à 60% des patients âgés présentaient un déclin cognitif [9–13], une étude japonaise rapportait chez des patients hémodialysés âgés de 65 ans et plus une incidence de la démence de 4,2%: maladie d'Alzheimer et démences vasculaires, L'incidence de la Démence vasculaire était 7,4 fois plus élevée que dans la population générale âgée [14]. Dans notre étude 6,8% des patients avaient des troubles cognitifs à type de troubles de sommeil, syndrome anxiodépressif et démence.

En l'absence de traitement curatif disponible, la prise en charge doit privilégier le contrôle des facteurs de risque vasculaire et la correction de l'anémie, afin de ralentir la progression du déclin cognitif Chez les dialysés [15].

Concernant les perturbations du métabolisme minéral et osseux, on note une prévalence élevée de l'ostéoporose et de l'ostéomalacie du fait de la carence fréquente et sévère en 25 OH vitamine D3. Ces atteintes osseuses exposent aux risques de fractures surtout que, souvent, dans ces tranches d'âge, s'associent diverses causes de chutes qu'il faut prévenir et/ou traiter: désordres neuromusculaires, troubles de l'équilibre, impotences diverses, troubles visuels, hypotension artérielle et causes cardiaques, interférences médicamenteuses [6]. Dans le cas de nos patients âgés dialysés, les troubles du métabolisme minéral et osseux étaient notés dans 71,8% des cas, il s'agissait d'hyperparathyroidie secondaire dans 62,5% des cas, hypoparathyroidie dans 6,2% des cas et ostéomalacie dans 3,1% des cas. Le traitement de nos patients était limité à la supplémentation calcique ainsi que la chélation du phosphore basé sur les chélateurs calciques, qui étaient utilisés dans 56,2% des cas.

De surcroît, l'anémie chez le patient hémodialysé âgé, est le plus souvent secondaire à une carence martiale du fait des pertes sanguines souvent occultes, souvent d'origine digestive. Celles-ci peuvent être aggravées par l'anticoagulation des séances, les divers traitements antithrombotiques, antiagrégants plaquettaires fréquemment utilisés. Les apports alimentaires insuffisants en fer ou des troubles de l'absorption de ce dernier peuvent également expliquer la carence martiale dans certains cas. Non traitée correctement, l'anémie peut altérer la qualité de vie et l'autonomie du patient. Elle peut également se compliquer de troubles cardiaques et d'atteinte des fonctions cognitives [16, 17]. Les cibles d'hémoglobine, les protocoles de supplémentation et de correction de cette anémie, ainsi que la réponse aux agents stimulant l'érythropoïèse sont les mêmes que pour les patients plus jeunes [18].

Dans notre série, 80% des patients étaient anémiques, avec une hémoglobine moyenne à 8,8±1,8. Cependant, seuls 33% de nos patients sont actuellement sous ASE. Ceci est clairement expliqué par le fait que ces médicaments sont coûteux et souvent inaccessibles, du fait que la plupart de nos patients sont de faibles niveaux socio-économiques. Ce qui rend la gestion des complications propres à la dialyse et celles inhérentes à la vieillesse une tâche délicate dans notre pratique quotidienne.

## Conclusion

Les sujets âgés en HDC représentent une population de patients fragiles. Ils nécessitent une surveillance particulière et régulière afin de prévenir certaines complications propres au sujet âgé et améliorer ainsi leur qualité de vie. L'efficacité du traitement par hémodialyse ne doit pas faire oublier l'importance du traitement préventif chez le Sujet âgés qui est basé sur le dépistage des maladies rénales et la sensibilisation des praticiens de première ligne et la meilleure coordination entre néphrologues et autres spécialistes afin d'instaurer à temps ces mesures préventives sinon curatives.
